# Guidelines for Corticosteroid use in Treatment of DMD

**DOI:** 10.15844/pedneurbriefs-30-3-4

**Published:** 2016-03

**Authors:** Vamshi K. Rao

**Affiliations:** 1 Division of Neurology, Department of Pediatrics, University of Nebraska Medical Center and Children's Hospital and Medical Center, Omaha, NE

**Keywords:** Corticosteroids, Guidelines, Duchenne muscular dystrophy

## Abstract

The guideline development subcommittee of the American Academy of Neurology has provided an update to the 2005 treatment guidelines for use of corticosteroids (CS) in Duchenne muscular dystrophy (DMD).

The guideline development subcommittee of the American Academy of Neurology has provided an update to the 2005 treatment guidelines for use of corticosteroids (CS) in Duchenne muscular dystrophy (DMD). These addressed questions of (i) Efficacy of CS related to survival, quality of life (QoL), motor function, scoliosis, pulmonary and cardiac function (ii) side effects of CS treatment (iii) comparison of prednisone (PS) and deflazacort (DF) wrt efficacy and adverse effects (AE) profile (iv) optimal dosing regimens of CS and (v) useful interventions for maximizing bone health health. Guidelines were developed based on medline and Cochrane database searches of relevant articles addressing the issue. Class I-III trials from the original guideline were included and fewer than 10 patient trials were excluded. 63 articles met the inclusion criteria of which 24 were graded class I-III.

Following are the recommendations based on the analysis: Prednisone (a) should be used for improving strength and may be useful for improved timed motor function (eg time to stand) (b) should be used to improve pulmonary function (c) may reduce need for scoliosis surgery and (d) may delay onset of cardiomyopathy by 18yrs of age. Deflazacort (a) improves strength and timed motor function and delayed loss of ambulation by 1.4-2.5 yrs (b) improves pulmonary function (c) reduces the need for scoliosis surgery (d) delays cardiomyopathy by 18yrs of age and (d) increased survival at 5 and 15 years.

When compared both PS and DF are equivalent in improving motor function but there is insufficient evidence to establish a difference in effect on cardiac function. PS is associated with increased weight gain in first years of treatment compared to increased risk of cataracts with DF.

PS 0.75/mg/kg/d is the preferred dosing regimen. PS 10mg/kg on the weekend is equally effective over 12 months but long term effect is not established. AE profile is similar with both regimens over 12 months of treatment. PS 0.3mg/kg/d has lesser in efficacy and fewer AE profile. PS 1.5mg/kg/d is equivalent to 0.75mg/kg/d but with more AE.

There is insufficient evidence to support or refute the following: (a) addition of calcifediol or alendronate for improving bone health on PS (b) benefit of bisphosphonates on survival in patients on CS (c) benefit of prednisone for survival (d) differences in efficacy of AE between daily, alternate day or intermittent regimens for prednisone or prednisolone (e) preferred dose of deflazacort or (f) effect of corticosteroids on QoL. [1]

COMMENTARY. It has often been the experience of many in the field treating DMD, of time spent highlighting the paucity of evidence regarding the choice of CS, dosing regimens and AE thereof when patients are newly diagnosed. This paper has tried to address such issues and provided updates to previous guidelines that make such decisions somewhat easier in the short term. It should be noted that choice of PS vs DF in the US is also influenced by availability, as DF needs to be shipped from overseas.

Further questions that need to be answered include the time of initiation of CS treatment, length of CS therapy, change in dosing over time as motor functions decline. Some of these questions are being dealt in an ongoing large multicenter trial titled “finding the optimum regimen for DMD (FOR-DMD)”, which is actively recruiting and the results of which are much awaited.

In the interim, I strongly support treating DMD with CS while counseling parents on the current evidence available regarding efficacy, dosing regimens and AE. Gaps in knowledge need to be presented during initiation of treatment. While there are clinical trials underway trying to ameliorate the genetic mutations in DMD, CS still remain the best available medication and the above mentioned gaps should not the deter initiation of such.
